# Association Between Cirrhosis and Acute or Severe Limb Ischemic Events Among Hospitalizations with Peripheral Artery Disease: A National Inpatient Sample Analysis

**DOI:** 10.3390/medicina62061147

**Published:** 2026-06-12

**Authors:** Oday Salman, Elie Bou Sanayeh, Hadi Itani, Chloe Lahoud, Chapman Wei, Ahmad Mustafa, Anaha Raghunathan, Elie Moussa, Martin Miguel I. Amor

**Affiliations:** 1 Department of Internal Medicine, Northwell, New Hyde Park, NY 11040, USA; osalman@northwell.edu (O.S.); ebousanayeh@northwell.edu (E.B.S.); hitani@northwell.edu (H.I.); clahoud@northwell.edu (C.L.); cwei4@northwell.edu (C.W.); amustafa3@northwell.edu (A.M.); araghuna2@student.touro.edu (A.R.); emoussa@northwell.edu (E.M.); 2 Northwell Cardiovascular Institute, Northwell, New Hyde Park, NY 11040, USA

**Keywords:** peripheral artery disease, cirrhosis, acute or severe limb ischemic events, national inpatient sample, thrombosis, propensity score matching, vascular outcomes

## Abstract

*Background and Objectives*: Acute or severe limb ischemic events are limb- and life-threatening complications of peripheral artery disease (PAD). Although cirrhosis is increasingly recognized as a systemic disorder associated with endothelial dysfunction, inflammation, and altered hemostatic balance, its relationship with coding-defined limb ischemic events among individuals with PAD remains poorly defined. We investigated whether cirrhosis is independently associated with coding-defined acute or severe limb ischemic events among hospitalized adults with PAD. *Materials and Methods*: We performed a retrospective discharge-level analysis of the National Inpatient Sample from 2016 to 2018. Adult hospitalizations with PAD were identified using ICD-10-CM diagnosis codes and stratified according to the presence or absence of cirrhosis. The primary outcome was coding-defined acute or severe limb ischemic events. Multivariable logistic regression was used to evaluate the association between cirrhosis and acute or severe limb ischemic events after adjustment for demographic and cardiovascular comorbidities. Propensity-score matching was additionally performed to balance baseline characteristics between hospitalizations with and without cirrhosis. *Results*: Among 276,702 hospitalizations with PAD, 5942 had cirrhosis. Before matching, cirrhosis was independently associated with higher odds of acute or severe limb ischemic events after multivariable adjustment (odds ratio [OR] 1.47, 95% confidence interval [CI] 1.35–1.60; *p* < 0.001). After 1:1 propensity-score matching, 5883 matched pairs were analyzed. The association between cirrhosis and acute or severe limb ischemic events persisted in the matched cohort, with cirrhosis remaining significantly associated with increased odds of these events (OR 1.41, 95% CI 1.24–1.60; *p* < 0.001). *Conclusions*: In this large discharge-level analysis of hospitalizations with PAD, cirrhosis was independently associated with higher odds of coding-defined acute or severe limb ischemic events. These findings suggest that cirrhosis may represent an underrecognized vascular risk amplifier in PAD. Further prospective studies are needed to validate this association, clarify underlying mechanisms, and determine whether cirrhosis may improve PAD risk stratification.

## 1. Introduction

Peripheral artery disease (PAD) is a major clinical manifestation of systemic atherosclerosis and a growing global health burden. In 2015, approximately 237 million adults were living with PAD worldwide, with the highest absolute counts reported in low- and middle-income regions [1]. In the United States, PAD affects over 8 million adults aged ≥ 40 years, leading to considerable morbidity, impaired quality of life, and high healthcare utilization [2].

Acute or severe limb ischemic events represent limb- and life-threatening complications of PAD. The 2024 ACC/AHA PAD guidelines list diabetes, smoking, chronic kidney disease (CKD), hypertension, and dyslipidemia as major conventional atherosclerotic risk amplifiers for adverse limb events, yet cirrhosis is notably absent from this list [3].

Liver cirrhosis, the end stage of diverse hepatic insults such as viral hepatitis, alcohol-related liver disease, and metabolic dysfunction-associated steatotic liver disease (MASLD), is increasingly recognized as a contributor to systemic vascular dysfunction [4]. Large population-based studies have demonstrated that patients with cirrhosis exhibit a significantly higher risk of developing PAD compared with those without liver disease, independent of traditional cardiovascular risk factors [5,6]. Globally, the prevalence of cirrhosis has risen in recent years, with approximately 2.05 million new cases reported in 2019 [7]. In the United States, the prevalence is estimated at 2.6%, with MASLD now accounting for the majority of cases, paralleling the epidemics of obesity and type 2 diabetes [8].

The pathophysiological link between cirrhosis, particularly MASLD-related cirrhosis, and PAD is multifactorial. Both conditions share a clustering of cardiometabolic risk factors, including adiposity, insulin resistance, type 2 diabetes, dyslipidemia, hypertension, and smoking, which synergistically promote atherosclerosis [1,3,8]. Beyond these shared risk factors, cirrhosis induces a complex and unique alteration in hemostatic balance, characterized by deficiencies in both procoagulant and anticoagulant factors, elevated levels of factor VIII and von Willebrand factor, thrombocytopenia with variable platelet function, impaired fibrinolysis, and heightened endothelial dysfunction and inflammation [9]. Collectively, these changes can result in a relative hypercoagulable state, predisposing to both bleeding and arteriovenous thrombotic complications [10,11].

Emerging evidence further suggests that the severity of hepatic dysfunction, particularly advanced fibrosis or decompensation, amplifies the risk of vascular complications, including PAD and its sequelae [5,6,12]. As the prevalence of MASLD and cirrhosis continues to rise, understanding the interplay between hepatic and vascular pathology is critical for optimizing risk stratification and management strategies in this high-risk population. We therefore hypothesized that cirrhosis is independently associated with higher odds of coding-defined acute or severe limb ischemic events among hospitalizations with PAD. Accordingly, we aimed to assess the independent association between cirrhosis and these events in hospitalized adults with PAD, while adjusting for established cardiometabolic risk factors.

## 2. Materials and Methods

We conducted a retrospective cohort analysis using the Nationwide Inpatient Sample (NIS) database for the years 2016–2018, the largest publicly available all-payer inpatient care database in the United States. The NIS is maintained by the Agency for Healthcare Research and Quality as part of the Healthcare Cost and Utilization Project (HCUP) and comprises a 20% stratified sample of all inpatient hospital discharges, representing approximately 97% of the US population. Because the NIS is a discharge-level database, the unit of analysis was hospitalization rather than individual patient. Therefore, repeated hospitalizations by the same patient could not be identified. This study was deemed exempt from Institutional Review Board (IRB) approval and informed consent requirements, as all data are de-identified and publicly available, in accordance with HCUP Data Use Agreement and federal regulations [13].

We included adult hospitalizations (age ≥ 18 years) with a diagnosis of PAD, identified using validated International Classification of Diseases Tenth Revision (ICD-10) codes. PAD was identified using ICD-10 codes I70.2–I70.7 (atherosclerosis of native arteries of extremities) and I73.9 (peripheral vascular disease, unspecified). Cirrhosis was defined by K74.60 (unspecified cirrhosis), K70.30 (alcoholic cirrhosis), K74.69 (other cirrhosis), and related codes. Acute or severe limb ischemic events were captured using ICD-10-CM codes I74.3 and I70.221–I70.229. Code I74.3 identifies embolism and thrombosis of arteries of the lower extremities, whereas I70.221–I70.229 identify atherosclerosis of native arteries of the extremities with rest pain or tissue loss. Therefore, the outcome should be interpreted as coding-defined acute or severe limb ischemic events captured using administrative diagnosis codes. This coding strategy may capture a spectrum of acute, acute-on-chronic, and severe limb-threatening ischemic presentations rather than purely embolic or thrombotic acute limb ischemia. All ICD-10 codes used to define study exposures, outcomes, and comorbidities are detailed in Supplementary Table S1.

Hospitalizations were excluded if they had a history of organ transplantation, pregnancy, cancer, age under 18 years, missing data, bleeding disorders, or thrombophilia to minimize confounding in the assessment of thrombosis or bleeding risk. Eligible hospitalizations were stratified into two cohorts based on the presence or absence of a diagnosis of liver cirrhosis, as defined by ICD-10 codes. The primary outcome was the rate of coding-defined acute or severe limb ischemic events among hospitalizations with cirrhosis compared with hospitalizations without cirrhosis, identified using established diagnostic codes.

### Statistical Analysis

A multi-step analytical approach was used to compare outcomes between the cirrhosis and non-cirrhosis cohorts. Baseline characteristics were summarized descriptively; continuous variables were reported using means with standard deviations and compared using *t*-tests or one-way analysis of variance (ANOVA), as appropriate, while categorical variables were reported as counts with percentages and compared using chi-square tests or Fisher’s exact tests, as appropriate.

To address potential confounding and improve balance in baseline characteristics, propensity-score matching was performed using a 1:1 greedy nearest-neighbor matching algorithm without replacement, with a caliper width of 0.2 standard deviations of the logit of the propensity score. The propensity score was estimated using logistic regression and included age, sex, smoking status, dyslipidemia, diabetes mellitus, obesity, chronic heart failure, hypertension, end-stage renal disease, coronary artery disease, atrial fibrillation, and prior cerebrovascular accident. Adequate overlap of propensity scores between hospitalizations with and without cirrhosis was confirmed.

After matching, covariate balance was assessed using standardized mean differences (SMDs), with values < 0.10 considered indicative of adequate balance. The matched cohorts were then compared for the primary outcome using univariate and multivariable logistic regression models, accounting for the matched design using robust standard errors clustered on matched pairs, to quantify effect sizes and adjust for any residual imbalance. Variables included in multivariable models were selected based on clinical relevance and prior literature on PAD and limb ischemic event risk factors.

Statistical significance was defined as a two-sided *p*-value < 0.05. All analyses were conducted using SPSS software (version 25; IBM Corp., Armonk, NY, USA).

## 3. Results

Baseline characteristics of the study cohort are summarized in Table 1. Among 276,702 hospitalizations with PAD, 270,760 had no cirrhosis and 5942 (2.1%) had cirrhosis. Compared with hospitalizations without cirrhosis, those with cirrhosis were younger (mean age 68.4 vs. 72.2 years; *p* < 0.001), more frequently male (58.9% vs. 52.5%; *p* < 0.001), and had higher prevalence of diabetes (50.0% vs. 43.8%; *p* < 0.001), chronic heart failure (31.0% vs. 27.9%; *p* < 0.001), and obesity (16.9% vs. 15.9%; *p* = 0.041). Conversely, hospitalizations with cirrhosis had lower rates of smoking (29.1% vs. 33.5%; *p* < 0.001), dyslipidemia (35.4% vs. 46.0%; *p* < 0.001), and prior CVA (7.9% vs. 11.9%; *p* < 0.001). Additional comorbidities, including hypertension, end-stage renal disease, and coronary artery disease, also differed significantly between groups (all *p* < 0.001).

After 1:1 propensity-score matching using age, sex, smoking status, dyslipidemia, diabetes mellitus, obesity, chronic heart failure, hypertension, end-stage renal disease, coronary artery disease, atrial fibrillation, and prior cerebrovascular accident, 5883 matched pairs of hospitalizations with and without cirrhosis were obtained (Table 2). Post-matching balance assessment using standardized mean differences (SMDs) demonstrated excellent covariate balance across baseline demographics and comorbidities, with all SMDs < 0.10, indicating well-balanced cohorts for key variables.

**Figure 1:**
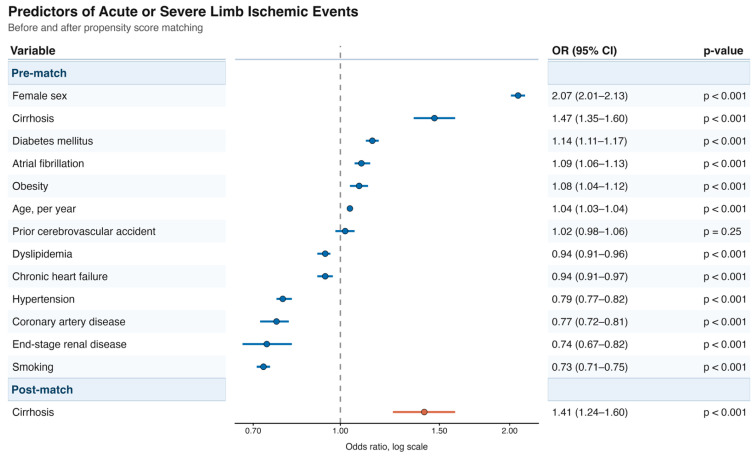
Multivariable Logistic Regression Identifying Predictors of Acute or Severe Limb Ischemic Events Before and After Propensity Score Matching. Values are shown as odds ratios with 95% confidence intervals and *p*-values. Blue markers indicate estimates from the pre-matched multivariable model, and the orange marker indicates the post-matched cirrhosis estimate. Horizontal bars represent 95% confidence intervals.

Multivariable logistic regression in the pre-matched cohort (Figure 1) demonstrated that cirrhosis was independently associated with increased odds of acute or severe limb ischemic events (OR 1.47, 95% CI 1.35–1.60; *p* < 0.001). Other variables independently associated with higher odds of acute or severe limb ischemic events included older age (OR 1.04 per year, 95% CI 1.03–1.04; *p* < 0.001), female sex (OR 2.07, 95% CI 2.01–2.13; *p* < 0.001), diabetes (OR 1.14, 95% CI 1.11–1.17; *p* < 0.001), obesity (OR 1.08, 95% CI 1.04–1.12; *p* < 0.001), and atrial fibrillation (OR 1.09, 95% CI 1.06–1.13; *p* < 0.001).

In the post-matched analysis (Figure 1) cirrhosis was significantly and independently associated with higher odds of acute or severe limb ischemic events (OR 1.41, 95% CI 1.24–1.60; *p* < 0.001), confirming the robustness of this relationship after adjustment for measured confounders.

## 4. Discussion

In this large cross-sectional analysis of more than 270,000 hospitalizations with PAD, cirrhosis was found to be independently associated with significantly higher odds of coding-defined acute or severe limb ischemic events. This association persisted even after propensity score matching and adjustment for major cardiometabolic risk factors, highlighting cirrhosis as a possible underrecognized risk factor for these events among hospitalizations with PAD. The inverse associations observed for several conventional cardiovascular risk factors in the pre-matched model should be interpreted cautiously and should not be considered protective. Because the analytic cohort was restricted to hospitalizations with PAD, conditioning on PAD-related hospitalization may introduce collider bias and distort associations between established vascular risk factors and limb ischemic outcomes. In addition, administrative coding may incompletely capture chronic comorbidities during complex admissions dominated by limb-threatening ischemia, infection, procedures, or cirrhosis-related complications. Patients with recognized cardiovascular risk factors may also differ in treatment intensity, statin or antiplatelet exposure, vascular surveillance, and outpatient follow-up, none of which can be fully assessed in the NIS. Therefore, these inverse associations likely reflect residual confounding, selection mechanisms, treatment patterns, and administrative coding limitations rather than true protective effects.

To our knowledge, this is the first study to specifically demonstrate an independent association between cirrhosis and coding-defined acute or severe limb ischemic events in a PAD population. Current 2024 ACC/AHA/Multisociety PAD guidelines delineate conventional risk amplifiers that modify PAD prognosis and ischemic limb outcomes, such as diabetes, smoking, CKD, and female sex [3]. However, these guidelines do not mention cirrhosis or chronic liver disease as either a risk factor or a risk enhancer for limb ischemic events, highlighting an area that may warrant further investigation.

### 4.1. Cirrhosis and Arterial Thrombosis

The role of cirrhosis in arterial thrombotic disease remains incompletely defined. Large cohort studies have shown a relatively consistent association with ischemic stroke, where cirrhosis independently increased stroke risk even after adjustment for conventional risk factors [14]. In contrast, the relationship between cirrhosis and myocardial infarction or coronary artery disease has been more equivocal. While some cohorts demonstrated increased risk of acute coronary syndrome [5], meta-analytic evidence has not shown a significant association between cirrhosis and myocardial infarction overall [15]. Our findings extend this heterogeneity to the peripheral circulation, demonstrating that cirrhosis is independently associated with coding-defined acute or severe limb ischemic events among hospitalizations with PAD.

This supports the concept that thrombotic risk in cirrhosis is not uniform across vascular beds, likely reflecting differences in local vascular physiology, endothelial function, and the impact of systemic inflammation. Meta-analyses and cohort studies indicate that the risk of arterial events may differ by cirrhosis etiology. For example, viral cirrhosis (hepatitis B or C) is associated with higher cardiovascular risk, while alcohol-related cirrhosis shows a weaker or null association [15].

Cirrhosis is clinically heterogeneous, and vascular risk may vary according to etiology, severity, and decompensation status. Alcohol-related liver disease, viral hepatitis, and MASLD-related cirrhosis may have distinct inflammatory, metabolic, endothelial, and thrombotic profiles. Excessive alcohol exposure, for example, has been linked to oxidative stress, inflammation, hypertension, prothrombotic states, coronary vasospasm, and arrhythmogenesis, supporting the concept that vascular risk may differ across liver disease etiologies [16]. However, administrative ICD-10 codes alone may incompletely capture cirrhosis phenotype, disease severity, and overlapping etiologies, particularly in discharge-level data without laboratory values, imaging findings, validated cirrhosis severity scores, medication exposure, or longitudinal hepatology follow-up. Therefore, the present findings should be interpreted as an overall association between cirrhosis and coding-defined limb ischemic events rather than as an etiology-specific or severity-specific estimate.

This further highlights the need for future patient-level studies with validated cirrhosis phenotyping to assess whether the association between cirrhosis and limb ischemic events differs by cirrhosis etiology, severity, or decompensation status. While the hypercoagulable state is a common feature, the clinical expression may be modulated by underlying liver disease etiology, comorbidities, and local vascular factors. This has direct implications for risk assessment, prophylaxis, and management in cirrhotic patients.

### 4.2. Shared Risk Factor Profile and Mechanistic Plausibility

The substantial overlap in risk factor profiles between PAD and cirrhosis, especially MASLD-related cirrhosis, provides compelling pathophysiologic plausibility for their observed association. MASLD has emerged as the leading cause of cirrhosis in the United States and is tightly linked to obesity, insulin resistance, type 2 diabetes, hypertension, and dyslipidemia, all known risk factors for PAD [17]. This convergence underscores the need to disentangle whether the increased PAD and limb ischemic event risk among cirrhotic patients is primarily driven by the systemic metabolic milieu or by cirrhosis-specific alterations in hemostatic and vascular function. Stratified analyses by cirrhosis etiology may therefore help clarify whether the relationship reflects the intrinsic consequences of cirrhosis itself, characterized by a delicate interplay between hypo- and hypercoagulability, endothelial dysfunction, and systemic inflammation [11,18], or merely the clustering of shared cardiometabolic risk factors.

### 4.3. Clinical Implications

Taken together, our findings suggest that cirrhosis may represent a previously underrecognized marker of higher risk for acute or severe limb ischemic events among hospitalizations with PAD. These findings have potential clinical implications but should be interpreted as hypothesis-generating. Our findings may support heightened clinical awareness in patients with PAD and cirrhosis, particularly during periods of acute hepatic decompensation or hospitalization, when coagulopathy and systemic inflammation may be exacerbated. Future studies should evaluate whether incorporating cirrhosis-related variables into PAD risk-stratification models improves identification of patients at higher risk for adverse limb outcomes. Until such data are available, cirrhosis should be viewed as a potential risk amplifier that warrants further prospective validation rather than immediate incorporation into PAD management algorithms.

### 4.4. Management Considerations and Safety Issues

Antithrombotic management in patients with cirrhosis requires a nuanced, individualized approach. Antiplatelet and anticoagulant therapy are generally considered safe in patients with compensated cirrhosis (Child–Pugh A/B), although direct oral anticoagulants remain contraindicated in severe hepatic impairment [19,20]. Severe thrombocytopenia remains a relative contraindication to both antiplatelet and anticoagulant use. Therefore, individualized careful assessment of bleeding risk, liver function status, and platelet count is essential before initiating therapy. Close clinical and laboratory monitoring is critical, particularly during periods of acute hepatic decompensation or when adjusting antithrombotic regimens.

### 4.5. Limitations

This study has several limitations that warrant careful consideration. First, the NIS is a discharge-level administrative database rather than a patient-level longitudinal cohort. Therefore, individual patients could not be followed over time, repeated hospitalizations by the same patient could not be identified, and readmissions or recurrent limb ischemic events could not be distinguished from index hospitalizations. Consequently, our findings should be interpreted as associations among PAD hospitalizations rather than estimates of patient-level incidence, recurrence, or longitudinal risk. In addition, the cross-sectional design precludes definitive causal inference, and the temporal relationship between cirrhosis diagnosis and acute or severe limb ischemic events could not be fully established. Second, reliance on administrative coding introduces the possibility of misclassification of both cirrhosis and limb ischemic events, as diagnostic accuracy could not be verified through clinical records or imaging data. Because ICD-10-CM codes I70.221–I70.229 may capture acute-on-chronic or chronic limb-threatening ischemia rather than purely embolic or thrombotic acute limb ischemia, outcome misclassification is possible. Third, although propensity score matching and multivariable adjustment were employed to minimize bias, residual confounding from unmeasured variables cannot be excluded. The NIS lacks granular PAD-specific clinical variables, including ankle-brachial index, vascular imaging findings, anatomic distribution of disease, wound severity, limb infection severity, outpatient vascular follow-up, medication exposure, and adherence to antiplatelet, anticoagulant, or statin therapy. Prior revascularization and procedure history may also be incompletely captured when they occurred before the index hospitalization. Therefore, residual confounding by PAD severity, treatment intensity, and outpatient vascular care cannot be excluded. Finally, our analysis was limited to in-hospital acute or severe limb ischemic events; therefore, we were unable to evaluate long-term outcomes, including post-discharge recurrence of limb ischemic events, mortality, or major adverse cardiovascular events.

Additionally, we did not perform etiology-specific or severity-specific subgroup analyses for cirrhosis. Although alcohol-related, viral, and MASLD-related cirrhosis may have different vascular and thrombotic risk profiles, robust stratification was not feasible with adequate clinical granularity in the NIS. The NIS is a discharge-level administrative database derived from hospital discharge abstracts and does not include laboratory values, imaging findings, medication exposure, validated cirrhosis severity scores, or longitudinal hepatology assessments. ICD-10 codes may identify selected cirrhosis etiologies or decompensating features, but they may incompletely capture the underlying cirrhosis phenotype, overlap across etiologic categories, and reflect acute complications documented during the index hospitalization rather than stable baseline cirrhosis severity. Therefore, additional stratification by cirrhosis etiology or decompensation status was not pursued because such analyses would have been vulnerable to exposure misclassification, unstable subgroup estimates, and limited clinical interpretability. Future patient-level studies with validated hepatology phenotyping should evaluate whether cirrhosis etiology and severity modify the relationship between cirrhosis and limb ischemic events in PAD.

Future prospective studies incorporating detailed clinical data and stratification by cirrhosis etiology are warranted to validate these findings, clarify underlying mechanisms, and determine whether the excess risk of limb ischemic events in cirrhosis reflects intrinsic hemostatic alterations, shared cardiometabolic exposures, or a combination of both. Such studies are essential to guide optimal surveillance, preventive and therapeutic strategies for patients with PAD and cirrhosis.

## 5. Conclusions

In this large, nationally representative discharge-level analysis of hospitalizations with PAD, cirrhosis was independently associated with higher odds of coding-defined acute or severe limb ischemic events, suggesting it may represent a potential marker of higher limb ischemic risk. This association likely reflects a combination of shared cardiometabolic exposures and cirrhosis-related hemostatic and inflammatory disturbances. These findings should be considered hypothesis-generating and require prospective validation before cirrhosis can be incorporated into PAD risk-stratification or management frameworks.

## Figures and Tables

**Table 1:** Baseline characteristics of hospitalizations with peripheral artery disease before propensity score matching.

Variables	No Cirrhosis (*n* = 270,760) ^1^	Cirrhosis (*n* = 5942) ^1^	*p*-Value
Age	72.16 ± 11.83	68.38 ± 10.52	<0.001
Female sex	128,641 (47.5)	2441 (41.1)	<0.001
Smoking	90,824 (33.5)	1731 (29.1)	<0.001
Dyslipidemia	124,640 (46.0)	2101 (35.4)	<0.001
Diabetes mellitus	118,628 (43.8)	2972 (50.0)	<0.001
Obesity	42,981 (15.9)	1002 (16.9)	0.041
Chronic heart failure	75,652 (27.9)	1844 (31.0)	<0.001
Hypertension	109,396 (40.4)	1875 (31.6)	<0.001
End-stage renal disease	24,702 (9.1)	740 (12.5)	<0.001
Coronary artery disease	18,726 (6.7)	212 (3.6)	<0.001
Atrial fibrillation	54,976 (20.3)	1159 (19.5)	0.134
Prior cerebrovascular accident	32,257 (11.9)	471 (7.9)	<0.001

^1^ Values are presented as mean ± SD or *n* (%).

**Table 2:** Baseline characteristics of hospitalizations with peripheral artery disease after propensity score matching.

Variables	No Cirrhosis (*n* = 5883) ^1^	Cirrhosis (*n* = 5883) ^1^	SMD ^2^
Age	68.58 ± 10.44	68.53 ± 10.39	0.005
Female sex	2426 (41.2)	2422 (41.2)	0.001
Smoking	1707 (29.0)	1706 (29.0)	0.001
Dyslipidemia	2091 (35.5)	2084 (35.4)	0.002
Diabetes mellitus	2956 (50.2)	2938 (49.9)	0.006
Obesity	974 (16.6)	977 (16.6)	0.001
Chronic heart failure	1792 (30.5)	1809 (30.7)	0.004
Hypertension	1868 (31.8)	1864 (31.7)	0.002
End-stage renal disease	679 (11.5)	715 (12.2)	0.022
Coronary artery disease	195 (3.3)	208 (3.5)	0.011
Atrial fibrillation	1120 (19.0)	1131 (19.2)	0.005
Prior cerebrovascular accident	444 (7.5)	456 (7.8)	0.011

^1^ Values are presented as mean ± SD or *n* (%). ^2^ Standardized mean differences (SMDs) were used to assess covariate balance; SMD < 0.10 indicates adequate balance.

## Data Availability

The NIS data are available from the HCUP Central Distributor to eligible users who complete the HCUP Data Use Agreement training and obtain appropriate data access. The authors are not permitted to redistribute the raw NIS data.

## Supplementary Materials

The following supporting information can be downloaded at: https://www.mdpi.com/article/10.3390/medicina62061147/s1, Table S1: ICD-10-CM codes used to define study exposures, outcomes, and comorbidities; Table S2: Logistic Regression Analysis of the Association Between Cirrhosis and Acute or Severe Limb Ischemic Events Before and After Propensity Score Matching.

## Abbreviations

ACC/AHA	American College of Cardiology/American Heart Association
CKD	Chronic Kidney Disease
CVA	Cerebrovascular Accident
MASLD	Metabolic Dysfunction–Associated Steatotic Liver Disease
OR	Odds Ratio
PAD	Peripheral Artery Disease

## References

[1] Song P., Rudan D., Zhu Y., Fowkes F.J.I., Rahimi K., Fowkes F.G.R., Rudan I. (2019). Global, regional, and national prevalence and risk factors for peripheral artery disease in 2015: An updated systematic review and analysis. Lancet Glob. Health.

[2] Criqui M.H., Matsushita K., Aboyans V., Hess C.N., Hicks C.W., Kwan T.W., McDermott M.M., Misra S., Ujueta F. (2021). Lower Extremity Peripheral Artery Disease: Contemporary Epidemiology, Management Gaps, and Future Directions: A Scientific Statement From the American Heart Association. Circulation.

[3] Gornik H.L., Aronow H.D., Goodney P.P., Arya S., Brewster L.P., Byrd L., Chandra V., Drachman D.E., Eaves J.M., Ehrman J.K. (2024). 2024 ACC/AHA/AACVPR/APMA/ABC/SCAI/SVM/SVN/SVS/SIR/VESS Guideline for the Management of Lower Extremity Peripheral Artery Disease: A Report of the American College of Cardiology/American Heart Association Joint Committee on Clinical Practice Guidelines. Circulation.

[4] Ginès P., Krag A., Abraldes J.G., Solà E., Fabrellas N., Kamath P.S. (2021). Liver cirrhosis. Lancet.

[5] Lin S.Y., Lin C.L., Lin C.C., Wang I.K., Hsu W.H., Kao C.H. (2018). Risk of acute coronary syndrome and peripheral arterial disease in chronic liver disease and cirrhosis: A nationwide population-based study. Atherosclerosis.

[6] Jepsen P., Tapper E.B., Deleuran T., Kazankov K., Askgaard G., Sørensen H.T., Vilstrup H., West J. (2021). Risk and Outcome of Venous and Arterial Thrombosis in Patients with Cirrhosis: A Danish Nation-wide Cohort Study. Hepatology.

[7] Xiao S., Xie W., Zhang Y., Lei L., Pan Y. (2023). Changing epidemiology of cirrhosis from 2010 to 2019: Results from the Global Burden Disease study 2019. Ann. Med..

[8] Chan W.K., Chuah K.H., Rajaram R.B., Lim L.L., Ratnasingam J., Vethakkan S.R. (2023). Metabolic Dysfunction-Associated Steatotic Liver Disease (MASLD): A State-of-the-Art Review. J. Obes. Metab. Syndr..

[9] Intagliata N.M., Northup P.G. (2015). Anticoagulant Therapy in Patients with Cirrhosis. Semin Thromb. Hemost..

[10] Lisman T., Hernandez-Gea V., Magnusson M., Roberts L., Stanworth S., Thachil J., Tripodi A. (2021). The concept of rebalanced hemostasis in patients with liver disease: Communication from the ISTH SSC working group on hemostatic management of patients with liver disease. J. Thromb. Haemost..

[11] Tripodi A., Mannucci P.M. (2011). The coagulopathy of chronic liver disease. N. Engl. J. Med..

[12] Younossi Z.M., de Avila L., Racila A., Nader F., Paik J., Henry L., Stepanova M. (2025). Prevalence and predictors of cirrhosis and portal hypertension in the United States. Hepatology.

[13] Healthcare Cost and Utilization Project (HCUP) (2016–2018). HCUP National Inpatient Sample (NIS), 2016–2018.

[14] Parikh N.S., Navi B.B., Schneider Y., Jesudian A., Kamel H. (2017). Association Between Cirrhosis and Stroke in a Nationally Representative Cohort. JAMA Neurol..

[15] Gu C., Dong L., Chai L., Tong Z., Gao F., Ageno W., Romeiro F.G., Qi X. (2025). Risk of Coronary Artery Disease in Patients with Liver Cirrhosis: A Systematic Review and Meta-analysis. J. Clin. Transl. Hepatol..

[16] Hagström H., Thiele M., Sharma R., Simon T.G., Roelstraete B., Söderling J., Sundström J., Ludvigsson J.F. (2023). Cardiovascular Outcomes in Patients with Biopsy-Proven Alcohol-Related Liver Disease. Clin. Gastroenterol. Hepatol..

[17] Cusi K., Abdelmalek M.F., Apovian C.M., Balapattabi K., Bannuru R.R., Barb D., Bardsley J.K., Beverly E.A., Corbin K.D., ElSayed N.A. (2025). Metabolic Dysfunction–Associated Steatotic Liver Disease (MASLD) in People with Diabetes: The Need for Screening and Early Intervention. A Consensus Report of the American Diabetes Association. Diabetes Care.

[18] Lisman T., Porte R.J. (2010). Rebalanced hemostasis in patients with liver disease: Evidence and clinical consequences. Blood.

[19] Tripodi A., Anstee Q.M., Sogaard K.K., Primignani M., Valla D.C. (2011). Hypercoagulability in cirrhosis: Causes and consequences. J. Thromb. Haemost..

[20] (2022). EASL Clinical Practice Guidelines on prevention and management of bleeding and thrombosis in patients with cirrhosis. J. Hepatol..

