# Supplementary material for: Association Between Cirrhosis and Acute or Severe Limb Ischemic Events Among Hospitalizations with Peripheral Artery Disease: A National Inpatient Sample Analysis
Source: Medicina (Kaunas). 2026 Jun 12;62(6):1147. doi: 10.3390/medicina62061147 (PMC13302823; doi:10.3390/medicina62061147)
Supplement: Supplementary file 1 [file medicina-62-01147-s001.zip › medicina-4336942-supplementary.pdf]

Supplementary Table S1. ICD-10-CM codes used to define study exposures, outcomes, and comorbidities.

Variable	ICD-10 Codes
Smoking	F17.200, Z87.891
Dyslipidemia	E78.0, E78.1, E78.2, E78.3, E78.4, E78.5
Diabetes Mellitus	E08, E09, E10, E11, E13
Obesity	E66.01, E66.09, E66.1, E66.2, E66.8, E66.9, Z68.30, Z68.31, Z68.32, Z68.33, Z68.34, Z68.35, Z68.36, Z68.37, Z68.38, Z68.39, Z68.41, Z68.42, Z68.43, Z68.44, Z68.45, Z68.54
Chronic Heart Failure	I11.0, I13.0, I09.81, I50.1, I50.20, I50.22, I50.30, I50.32, I50.40, I50.42, I50.810, I50.812, I50.814, I50.82, I50.83, I50.84, I50.89, I50.9, I51.81, I97.130, Z95.812
Hypertension	I10
End-Stage Renal Disease	N18.6
Coronary Artery Disease	I25.10, I25.2, I25.8, I25.9
Atrial Fibrillation	I48
Prior Cerebrovascular Accident	Z86.73
Peripheral Artery Disease	I70.2, I70.3, I70.4, I70.5, I70.6, I70.7, I73.9
Cirrhosis	K74.1, K74.2, K74.3, K74.4, K74.5, K74.60, K74.69, K70.2, K70.30, K70.31, K71.7
Acute or severe limb ischemic events	I74.3, I70.221-I70.229

Supplementary Table S2. Logistic Regression Analysis of the Association Between Cirrhosis and Acute or Severe Limb Ischemic Events Before and After Propensity Score Matching.

Variables	Odds Ratio (OR)	95% CI (Lower–Upper)	p-value
Cirrhosis	1.47	1.35–1.60	<0.001
Age	1.04	1.03–1.04	<0.001
Female sex	2.07	2.01–2.13	<0.001
Smoking	0.73	0.71–0.75	<0.001
Dyslipidemia	0.94	0.91–0.96	<0.001
Diabetes mellitus	1.14	1.11–1.17	<0.001
Obesity	1.08	1.04–1.12	<0.001
Chronic heart failure	0.94	0.91–0.97	<0.001
Hypertension	0.79	0.77–0.82	<0.001
End-stage renal disease	0.74	0.67–0.82	<0.001
Coronary artery disease	0.77	0.72–0.81	<0.001
Atrial fibrillation	1.09	1.06–1.13	<0.001
Prior cerebrovascular accident	1.02	0.98–1.06	0.25
**Logistic Regression Post-Matching (n=11,766)**			
Cirrhosis	1.41	1.24–1.60	<0.001
